# Supplementary material for: Trends of adult height in India from 1998 to 2015: Evidence from the National Family and Health Survey
Source: PLoS One. 2021 Sep 17;16(9):e0255676. doi: 10.1371/journal.pone.0255676 (PMC8448320; doi:10.1371/journal.pone.0255676)
Supplement: S1 Table — (DOCX) [file pone.0255676.s001.docx]

# Supportive information (S1 Table)

| **S1 Table Distribution of mean height of Indian according to the age group** | | | | | | | |
| --- | --- | --- | --- | --- | --- | --- | --- |
| **Gender and age groups** | **NFHS rounds** | **Mean height** | **Coefficient** | **Robust Std. Err.** | **P-value** | **[95% Conf. Interval]** | |
| **15-25 Years** | | | | | | | |
| Men | NFHS-4 | 163.38 | -1.10 | 0.11 | 0.001 | -1.31 | -0.99 |
|  | NFHS-3 | 164.48 |  |  |  |  |  |
| Women | NFHS-4 | 151.83 | -0.12 | 0.06 | 0.051 | -0.24 | 0.00 |
|  | NFHS-3 | 151.95 | 0.84 | 0.08 | 0.0001 | 0.69 | 0.99 |
|  | NFHS-2 | 151.11 |  |  |  |  |  |
| **26-50 Years** | | | | | | | |
| Men | NFHS-4 | 163.68 | -0.86 | 0.09 | 0.001 | -1.03 | -0.69 |
|  | NFHS-3 | 164.54 |  |  |  |  |  |
| Women | NFHS-4 | 151.97 | 0.13 | 0.05 | 0.015 | 0.02 | 0.23 |
|  | NFHS-3 | 151.85 | 0.55 | 0.06 | 0.001 | 0.43 | 0.67 |
|  | NFHS-2 | 151.30 |  |  |  |  |  |
