# Supplementary material for: Trends of adult height in India from 1998 to 2015: Evidence from the National Family and Health Survey
Source: PLoS One. 2021 Sep 17;16(9):e0255676. doi: 10.1371/journal.pone.0255676 (PMC8448320; doi:10.1371/journal.pone.0255676)
Supplement: S2 Table — (DOCX) [file pone.0255676.s002.docx]

# Supportive information (S2 Table)

| **S2 Table Distribution of mean height of men and women according the age group and religion, rounds NFHS-3 and NFHS-4** | | | | | | | | | | | | | | |  |
| --- | --- | --- | --- | --- | --- | --- | --- | --- | --- | --- | --- | --- | --- | --- | --- |
| **Religion** | **Men** | | | | | | | **Women** | | | | | | |  |
|  | **NFHS-4** | **NFHS-3** | **Coef.** | **Robust Std. Err.** | **P-value** | **[95% Conf. Interval]** | | **NFHS-4** | **NFHS-3** | **Coef.** | **Robust Std. Err.** | **P-value** | **[95% Conf. Interval]** | |  |
| 15-25 years | | | | | | | | | | | | | | | |
| Hindu | 163.39 | 164.39 | -1.00 | 0.12 | 0.001 | -1.24 | -0.77 | 151.75 | 151.85 | -0.10 | 0.07 | 0.139 | -0.23 | 0.03 |  |
| Muslim | 163.18 | 164.46 | -1.27 | 0.31 | 0.001 | -1.89 | -0.66 | 151.94 | 152.11 | -0.17 | 0.15 | 0.277 | -0.47 | 0.13 |  |
| Christian | 162.59 | 163.49 | -0.90 | 0.62 | 0.145 | -2.11 | 0.31 | 152.05 | 152.28 | -0.23 | 0.31 | 0.449 | -0.83 | 0.37 |  |
| Sikh | 167.45 | 169.39 | -1.94 | 0.53 | 0.001 | -2.99 | -0.89 | 155.27 | 155.93 | -0.66 | 0.25 | 0.009 | -1.16 | -0.16 |  |
| Buddhist/neo-Buddhist | 161.43 | 163.34 | -1.91 | 0.87 | 0.029 | -3.62 | -0.20 | 151.39 | 151.00 | 0.40 | 0.56 | 0.478 | -0.70 | 1.49 |  |
| Jain | 165.31 | 169.61 | -4.30 | 1.87 | 0.024 | -8.02 | -0.58 | 155.45 | 153.74 | 1.72 | 0.85 | 0.044 | 0.05 | 3.38 |  |
| Jewish | 167.20 |  |  |  |  |  |  | 150.65 | 164.89 | -14.24 | 1.42 | 0.001 | -17.71 | -10.77 |  |
| Parsi/Zoroastrian | 156.62 | 165.50 | -8.88 | 1.93 | 0.019 | -15.02 | -2.75 | 151.50 | 154.72 | -3.23 | 2.73 | 0.242 | -8.69 | 2.24 |  |
| no religion | 154.04 | 162.43 | -8.39 | 2.27 | 0.002 | -13.17 | -3.62 | 148.29 | 146.85 | 1.44 | 2.07 | 0.488 | -2.66 | 5.54 |  |
| 26-50 Years | | | | | | | | | | | | | | | |
| Hindu | 163.59 | 164.44 | -0.84 | 0.09 | 0.001 | -1.03 | -0.66 | 151.83 | 151.74 | 0.09 | 0.06 | 0.105 | -0.02 | 0.20 |  |
| Muslim | 163.81 | 164.57 | -0.76 | 0.23 | 0.001 | -1.22 | -0.31 | 152.26 | 152.04 | 0.22 | 0.12 | 0.062 | -0.01 | 0.46 |  |
| Christian | 163.94 | 164.22 | -0.28 | 0.40 | 0.490 | -1.07 | 0.51 | 152.64 | 152.05 | 0.58 | 0.24 | 0.017 | 0.10 | 1.06 |  |
| Sikh | 168.31 | 170.48 | -2.18 | 0.38 | 0.001 | -2.93 | -1.42 | 156.08 | 155.71 | 0.36 | 0.19 | 0.055 | -0.01 | 0.74 |  |
| Buddhist/neo-Buddhist | 161.79 | 163.31 | -1.52 | 0.67 | 0.024 | -2.83 | -0.20 | 151.02 | 149.91 | 1.11 | 0.38 | 0.003 | 0.37 | 1.85 |  |
| Jain | 166.91 | 168.65 | -1.74 | 1.10 | 0.117 | -3.92 | 0.44 | 153.29 | 154.05 | -0.76 | 0.53 | 0.149 | -1.80 | 0.27 |  |
| Jewish | 159.70 | 163.73 | -4.03 | 0.40 | 0.002 | -5.31 | -2.74 | 149.81 | 155.57 | -5.76 | 2.48 | 0.059 | -11.83 | 0.31 |  |
| Parsi/Zoroastrian | 163.66 | 163.52 | 0.14 | 3.39 | 0.967 | -6.77 | 7.06 | 151.83 | 146.10 | 5.73 | 1.36 | 0.001 | 3.03 | 8.44 |  |
| No religion | 163.59 | 164.44 | -0.84 | 0.09 | 0.001 | -1.03 | -0.66 | 150.56 | 150.30 | 0.26 | 2.06 | 0.901 | -3.82 | 4.33 |  |
