# Supplementary material for: Trends of adult height in India from 1998 to 2015: Evidence from the National Family and Health Survey
Source: PLoS One. 2021 Sep 17;16(9):e0255676. doi: 10.1371/journal.pone.0255676 (PMC8448320; doi:10.1371/journal.pone.0255676)
Supplement: S3 Table — (DOCX) [file pone.0255676.s003.docx]

# Supportive information (S3 Table)

| **S3 Table Distribution of mean height of women according to religion, rounds NFHS-2 and NFHS-3** | | | | | | | |
| --- | --- | --- | --- | --- | --- | --- | --- |
| **Religion** | **NFHS-2** | **NFHS-3** | **Coef.** | **Robust Std. Err.** | **P-value** | **[95% Conf. Interval]** | |
| 15-25 years | | | | | | | |
| Hindu | 151.01 | 151.85 | 0.83 | 0.08 | 0.001 | 0.67 | 1.00 |
| Muslim | 151.37 | 152.11 | 0.74 | 0.21 | 0.001 | 0.33 | 1.14 |
| Christian | 151.56 | 152.28 | 0.72 | 0.40 | 0.074 | -0.07 | 1.50 |
| Sikh | 154.56 | 155.93 | 1.36 | 0.38 | 0.001 | 0.62 | 2.10 |
| Buddhist/neo-Buddhist | 150.06 | 151.00 | 0.94 | 0.71 | 0.189 | -0.46 | 2.34 |
| Jain | 152.99 | 153.74 | 0.75 | 1.01 | 0.457 | -1.24 | 2.74 |
| no religion | 149.51 | 154.72 | 5.22 | 6.69 | 0.479 | -13.35 | 23.79 |
| other | 149.33 | 146.85 | -2.48 | 2.05 | 0.236 | -6.68 | 1.72 |
| 26-50 years | | | | | | | |
| Hindu | 151.16 | 151.74 | 0.59 | 0.07 | 0.001 | 0.46 | 0.72 |
| Muslim | 151.56 | 152.04 | 0.48 | 0.15 | 0.001 | 0.19 | 0.77 |
| Christian | 152.24 | 152.05 | -0.19 | 0.30 | 0.527 | -0.77 | 0.40 |
| Sikh | 155.25 | 155.71 | 0.47 | 0.26 | 0.070 | -0.04 | 0.97 |
| Buddhist/neo-Buddhist | 149.88 | 149.91 | 0.03 | 0.46 | 0.950 | -0.87 | 0.93 |
| Jain | 153.75 | 154.05 | 0.31 | 0.54 | 0.574 | -0.76 | 1.38 |
| Jewish | 146.06 | 155.57 | 9.51 | 4.32 | 0.115 | -4.25 | 23.27 |
| Parsi/Zoroastrian | 147.77 | 146.10 | -1.67 | 2.24 | 0.484 | -7.16 | 3.82 |
| no religion | 150.03 | 150.30 | 0.27 | 2.17 | 0.902 | -4.08 | 4.62 |
| other | 148.94 | 150.66 | 1.72 | 0.76 | 0.025 | 0.21 | 3.22 |
