# Supplementary material for: Trends of adult height in India from 1998 to 2015: Evidence from the National Family and Health Survey
Source: PLoS One. 2021 Sep 17;16(9):e0255676. doi: 10.1371/journal.pone.0255676 (PMC8448320; doi:10.1371/journal.pone.0255676)
Supplement: S4 Table — (DOCX) [file pone.0255676.s004.docx]

# Supportive information (S4 Table)

| **S4 Table Distribution of mean height of women according to the type of caste or tribe, rounds NFHS-2 and NFHS-3** | | | | | | | |
| --- | --- | --- | --- | --- | --- | --- | --- |
| **Type of caste or tribe** | **NFHS-2** | **NFHS-3** | **Coef.** | **Robust Std. Err.** | **P-value** | **[95% Conf. Interval]** | |
| 15 -25 years | | | | | | | |
| Scheduled caste | 150.10 | 150.81 | 0.71 | 0.15 | 0.001 | 0.42 | 1.00 |
| Scheduled tribe | 150.84 | 151.13 | 0.29 | 0.20 | 0.150 | -0.10 | 0.69 |
| Other backward class | 151.07 | 151.97 | 0.90 | 0.12 | 0.001 | 0.66 | 1.14 |
| None of them | 151.83 | 152.99 | 1.16 | 0.13 | 0.001 | 0.91 | 1.40 |
| 26-50 years | | | | | | | |
| Scheduled caste | 150.33 | 150.66 | 0.34 | 0.12 | 0.005 | 0.10 | 0.57 |
| Scheduled tribe | 150.74 | 151.27 | 0.53 | 0.17 | 0.002 | 0.19 | 0.86 |
| Other backward class | 151.06 | 151.77 | 0.72 | 0.10 | 0.001 | 0.53 | 0.91 |
| None of them | 152.08 | 152.78 | 0.70 | 0.09 | 0.001 | 0.51 | 0.88 |
