# Supplementary material for: Trends of adult height in India from 1998 to 2015: Evidence from the National Family and Health Survey
Source: PLoS One. 2021 Sep 17;16(9):e0255676. doi: 10.1371/journal.pone.0255676 (PMC8448320; doi:10.1371/journal.pone.0255676)
Supplement: S5 Table — (DOCX) [file pone.0255676.s005.docx]

# Supportive information (S5 Table)

| **S5 Table Distribution of mean height of men and women according to the type of caste or tribe, round NFHS-3 and NFHS-4** | | | | | | | | | | | | | | | |
| --- | --- | --- | --- | --- | --- | --- | --- | --- | --- | --- | --- | --- | --- | --- | --- |
|  | **Men** | | | | | | | **Women** | | | | | | | |
| **Type of caste or tribe** | **NFHS-4** | **NFHS-3** | **Coef.** | **Robust Std. Err.** | **P-value** | **[95% Conf. Interval]** | | **NFHS-4** | **NFHS-3** | **Coef.** | **Robust Std. Err.** | **P-value** | **[95% Conf. Interval]** | |  |
| 15 -25 years | | | | | | | |  | | | | | | | |
| Scheduled caste | 162.44 | 163.19 | -0.75 | 0.21 | 0.001 | -1.16 | -0.35 | 150.96 | 150.81 | 0.15 | 0.11 | 0.179 | -0.07 | 0.36 |  |
| Scheduled tribe | 161.25 | 162.19 | -0.94 | 0.30 | 0.002 | -1.53 | -0.35 | 150.71 | 151.13 | -0.42 | 0.16 | 0.007 | -0.73 | -0.12 |  |
| Other backward class | 163.57 | 164.46 | -0.89 | 0.16 | 0.001 | -1.20 | -0.58 | 151.95 | 151.97 | -0.02 | 0.09 | 0.838 | -0.20 | 0.16 |  |
| None of them | 164.75 | 166.17 | -1.41 | 0.24 | 0.001 | -1.88 | -0.95 | 152.87 | 152.99 | -0.12 | 0.11 | 0.254 | -0.33 | 0.09 |  |
| 26-50 years | | | | | | | |  |  |  |  |  |  |  |  |
| Scheduled caste | 162.54 | 162.97 | -0.43 | 0.16 | 0.007 | -0.75 | -0.12 | 151.16 | 150.66 | 0.50 | 0.10 | 0.001 | 0.31 | 0.69 |  |
| Scheduled tribe | 161.80 | 162.58 | -0.78 | 0.24 | 0.001 | -1.26 | -0.30 | 151.22 | 151.27 | -0.05 | 0.13 | 0.671 | -0.30 | 0.19 |  |
| Other backward class | 163.80 | 164.68 | -0.88 | 0.12 | 0.001 | -1.11 | -0.64 | 152.01 | 151.77 | 0.23 | 0.08 | 0.002 | 0.08 | 0.38 |  |
| None of them | 165.12 | 165.95 | -0.84 | 0.17 | 0.001 | -1.17 | -0.50 | 152.82 | 152.78 | 0.04 | 0.08 | 0.657 | -0.12 | 0.19 |  |
