# Supplementary material for: Trends of adult height in India from 1998 to 2015: Evidence from the National Family and Health Survey
Source: PLoS One. 2021 Sep 17;16(9):e0255676. doi: 10.1371/journal.pone.0255676 (PMC8448320; doi:10.1371/journal.pone.0255676)
Supplement: S6 Table — (DOCX) [file pone.0255676.s006.docx]

# Supportive information (S6 Table)

| **S6 Table Distribution of mean height of men and women according to the residence, NFHS-4 and NFHS-3** | | | | | | | |
| --- | --- | --- | --- | --- | --- | --- | --- |
| **Type of place or residence** | **NFHS-4** | **NFHS-3** | **Coefficient** | **Robust Std. Err.** | **P-value** | **[95% Conf. Interval]** | |
| **Men** | | | | | | | |
| 15-25 Years | | | | | | | |
| Urban | 164.04 | 165.42 | -1.38 | 0.21 | 0.001 | -1.79 | -0.97 |
| Rural | 163.03 | 163.95 | -0.92 | 0.13 | 0.001 | -1.17 | -0.67 |
| 26-50 Years | | | | | | | |
| Urban | 164.23 | 165.22 | -0.99 | 0.15 | 0.001 | -1.28 | -0.69 |
| Rural | 163.37 | 164.19 | -0.81 | 0.10 | 0.001 | -1.02 | -0.61 |
| **Women** | | | | | | | |
| 15-25 Years | | | | | | | |
| Urban | 152.49 | 152.69 | -0.21 | 0.11 | 0.065 | -0.43 | 0.01 |
| Rural | 151.54 | 151.63 | -0.10 | 0.07 | 0.171 | -0.24 | 0.04 |
| 26-50 Years | | | | | | | |
| Urban | 152.56 | 152.35 | 0.20 | 0.09 | 0.024 | 0.03 | 0.38 |
| Rural | 151.66 | 151.61 | 0.06 | 0.06 | 0.379 | -0.07 | 0.18 |
