# Supplementary material for: Trends of adult height in India from 1998 to 2015: Evidence from the National Family and Health Survey
Source: PLoS One. 2021 Sep 17;16(9):e0255676. doi: 10.1371/journal.pone.0255676 (PMC8448320; doi:10.1371/journal.pone.0255676)
Supplement: S7 Table — (DOCX) [file pone.0255676.s007.docx]

# Supportive information (S7 Table)

| **S7 Table Distribution of mean height of women according to the residence, NFHS-2 and NFHS-3** | | | | | | | |
| --- | --- | --- | --- | --- | --- | --- | --- |
| **Type of place or residence** | **NFHS-2** | **NFHS-3** | **Coefficient** | **Robust Std. Err.** | **P-value** | **[95% Conf. Interval]** | |
| 15-25 Years | | | | | | | |
| Urban | 151.48 | 152.69 | 1.22 | 0.14 | 0.001 | 0.95 | 1.49 |
| Rural | 151.02 | 151.63 | 0.62 | 0.09 | 0.001 | 0.44 | 0.80 |
| 26-50 Years | | | | | | | |
| Urban | 151.67 | 152.35 | 0.69 | 0.11 | 0.001 | 0.47 | 0.90 |
| Rural | 151.15 | 151.61 | 0.46 | 0.08 | 0.001 | 0.30 | 0.61 |
