# Supplementary material for: Trends of adult height in India from 1998 to 2015: Evidence from the National Family and Health Survey
Source: PLoS One. 2021 Sep 17;16(9):e0255676. doi: 10.1371/journal.pone.0255676 (PMC8448320; doi:10.1371/journal.pone.0255676)
Supplement: S8 Table — (DOCX) [file pone.0255676.s008.docx]

# Supportive information (S8 Table)

| **S8 Table Distribution of mean height of men and women according to the wealth index, NFHS-4 and NFHS-3** | | | | | | | | | | | | | | | |
| --- | --- | --- | --- | --- | --- | --- | --- | --- | --- | --- | --- | --- | --- | --- | --- |
|  | **Men** | | | | | | | **Women** | | | | | | | |
| Wealth Index | NFHS-4 | NFHS-3 | **Coef.** | **Robust Std. Err.** | **P-value** | **[95% Conf. Interval]** | | NFHS-4 | NFHS-3 | **Coef.** | **Robust Std. Err.** | **P-value** | **[95% Conf. Interval]** | |  |
| 15-25 Years | | | | | | | |  |  |  |  |  |  |  |  |
| Poorest | 160.18 | 161.61 | -1.43 | 0.23 | 0.001 | -1.89 | -0.97 | 149.74 | 150.37 | -0.63 | 0.12 | 0.001 | -0.87 | -0.40 |  |
| Poorer | 162.14 | 162.99 | -0.85 | 0.21 | 0.001 | -1.25 | -0.44 | 150.93 | 150.88 | 0.04 | 0.10 | 0.680 | -0.16 | 0.24 |  |
| Middle | 163.39 | 163.84 | -0.44 | 0.20 | 0.025 | -0.83 | -0.06 | 151.86 | 151.69 | 0.17 | 0.10 | 0.097 | -0.03 | 0.36 |  |
| Richer | 164.69 | 165.40 | -0.71 | 0.21 | 0.001 | -1.11 | -0.30 | 152.73 | 152.50 | 0.22 | 0.10 | 0.029 | 0.02 | 0.42 |  |
| Richest | 165.77 | 167.32 | -1.55 | 0.24 | 0.001 | -2.03 | -1.07 | 154.07 | 154.13 | -0.06 | 0.11 | 0.571 | -0.28 | 0.15 |  |
| 26-50 Years | | | | | | | |  | | | | | | | |
| Poorest | 161.06 | 162.35 | -1.29 | 0.18 | 0.001 | -1.64 | -0.93 | 150.13 | 150.69 | -0.57 | 0.10 | 0.001 | -0.76 | -0.37 |  |
| Poorer | 162.39 | 163.34 | -0.95 | 0.15 | 0.001 | -1.25 | -0.65 | 151.03 | 150.93 | 0.10 | 0.09 | 0.240 | -0.07 | 0.27 |  |
| Middle | 163.48 | 164.18 | -0.70 | 0.15 | 0.001 | -0.99 | -0.41 | 151.76 | 151.56 | 0.20 | 0.09 | 0.020 | 0.03 | 0.37 |  |
| Richer | 164.49 | 165.02 | -0.53 | 0.15 | 0.001 | -0.82 | -0.25 | 152.59 | 152.09 | 0.51 | 0.08 | 0.001 | 0.34 | 0.67 |  |
| Richest | 165.87 | 167.01 | -1.15 | 0.18 | 0.001 | -1.50 | -0.79 | 153.86 | 153.55 | 0.31 | 0.09 | 0.001 | 0.14 | 0.49 |  |
