# Supplementary material for: Trends of adult height in India from 1998 to 2015: Evidence from the National Family and Health Survey
Source: PLoS One. 2021 Sep 17;16(9):e0255676. doi: 10.1371/journal.pone.0255676 (PMC8448320; doi:10.1371/journal.pone.0255676)
Supplement: S9 Table — (DOCX) [file pone.0255676.s009.docx]

# Supportive information (S9 Table)

| **S9 Table State wise distribution of mean height of women according to age group, NFHS-3 and NFHS-2** | | | | | | | | | | | | | | | |
| --- | --- | --- | --- | --- | --- | --- | --- | --- | --- | --- | --- | --- | --- | --- | --- |
|  | 15 to 25 Years | | | | | | Age group 26 to 50 years | | | | | | | | |
| **State** | **NFHS-2** | **NFHS-3** | **Coef.** | **Robust Std. Err.** | **P- value** | **[95% Conf. Interval]** | | **NFHS-2** | **NFHS-3** | **Coef.** | **Robust Std. Err.** | **P- value** | **[95% Conf. Interval]** | |  |
| **India** | **151.11** | **151.95** | **0.84** | **0.08** | **0.001** | **0.69** | **0.99** | **151.30** | **151.85** | **0.55** | **0.06** | **0.001** | **0.43** | **0.67** |  |
| Andhra Pradesh | 151.38 | 152.01 | 0.62 | 0.25 | 0.012 | 0.14 | 1.11 | 151.03 | 151.54 | 0.50 | 0.19 | 0.010 | 0.12 | 0.88 |  |
| Arunachal Pradesh | 150.65 | 150.41 | -0.24 | 0.42 | 0.571 | -1.07 | 0.59 | 150.94 | 151.00 | 0.06 | 0.39 | 0.885 | -0.72 | 0.83 |  |
| Assam | 149.61 | 150.46 | 0.85 | 0.39 | 0.032 | 0.07 | 1.62 | 149.91 | 150.55 | 0.64 | 0.32 | 0.045 | 0.01 | 1.27 |  |
| Bihar | 149.22 | 150.08 | 0.86 | 0.23 | 0.001 | 0.41 | 1.31 | 149.61 | 150.51 | 0.90 | 0.21 | 0.001 | 0.49 | 1.31 |  |
| Chhattisgarh | NA | 151.64 | NA | NA | NA | NA | NA | NA | 151.20 | NA | NA | NA | NA | NA |  |
| Goa | 150.53 | 152.87 | 2.34 | 0.47 | 0.001 | 1.41 | 3.27 | 151.95 | 152.43 | 0.48 | 0.36 | 0.182 | -0.23 | 1.19 |  |
| Gujarat | 151.85 | 153.04 | 1.20 | 0.30 | 0.001 | 0.61 | 1.78 | 151.78 | 152.40 | 0.63 | 0.25 | 0.013 | 0.14 | 1.12 |  |
| Haryana | 153.66 | 154.80 | 1.14 | 0.31 | 0.001 | 0.52 | 1.76 | 154.55 | 154.95 | 0.40 | 0.27 | 0.140 | -0.13 | 0.93 |  |
| Himachal Pradesh | 152.56 | 153.88 | 1.32 | 0.33 | 0.001 | 0.66 | 1.98 | 152.76 | 153.60 | 0.84 | 0.22 | 0.001 | 0.41 | 1.27 |  |
| Jammu and Kashmir | 153.42 | 154.96 | 1.54 | 0.37 | 0.001 | 0.81 | 2.27 | 153.44 | 154.26 | 0.82 | 0.24 | 0.001 | 0.34 | 1.30 |  |
| Jharkhand | NA | 149.68 | NA | NA | NA | NA | NA | NA | 150.03 | NA | NA | NA | NA | NA |  |
| Karnataka | 152.00 | 152.74 | 0.74 | 0.27 | 0.008 | 0.20 | 1.28 | 151.98 | 152.57 | 0.59 | 0.21 | 0.005 | 0.18 | 1.01 |  |
| Kerala | 153.63 | 154.09 | 0.46 | 0.36 | 0.204 | -0.25 | 1.18 | 152.38 | 152.61 | 0.23 | 0.25 | 0.356 | -0.27 | 0.74 |  |
| Madhya Pradesh | 151.57 | 152.44 | 0.87 | 0.24 | 0.001 | 0.41 | 1.34 | 151.76 | 152.75 | 0.99 | 0.18 | 0.001 | 0.63 | 1.35 |  |
| Maharashtra | 151.27 | 152.41 | 1.14 | 0.25 | 0.001 | 0.64 | 1.63 | 151.40 | 151.64 | 0.24 | 0.19 | 0.202 | -0.13 | 0.61 |  |
| Manipur | 151.03 | 151.99 | 0.96 | 0.33 | 0.001 | 0.30 | 1.62 | 151.59 | 151.86 | 0.27 | 0.29 | 0.361 | -0.31 | 0.85 |  |
| Meghalaya | 150.42 | 148.15 | -2.27 | 0.78 | 0.004 | -3.80 | -0.73 | 150.81 | 149.42 | -1.39 | 0.80 | 0.082 | -2.97 | 0.18 |  |
| Mizoram | 151.32 | 151.62 | 0.30 | 0.46 | 0.516 | -0.61 | 1.21 | 151.65 | 151.77 | 0.12 | 0.37 | 0.738 | -0.60 | 0.85 |  |
| Nagaland | 150.70 | 152.20 | 1.51 | 0.50 | 0.003 | 0.52 | 2.49 | 151.91 | 152.93 | 1.02 | 0.39 | 0.009 | 0.25 | 1.79 |  |
| Delhi | 152.03 | 153.31 | 1.29 | 0.50 | 0.010 | 0.31 | 2.26 | 152.65 | 154.16 | 1.51 | 0.38 | 0.001 | 0.77 | 2.25 |  |
| Odisha | 150.53 | 150.88 | 0.35 | 0.26 | 0.182 | -0.16 | 0.87 | 150.49 | 150.97 | 0.47 | 0.21 | 0.024 | 0.06 | 0.89 |  |
| Punjab | 154.18 | 154.76 | 0.58 | 0.34 | 0.085 | -0.08 | 1.24 | 154.59 | 154.68 | 0.09 | 0.25 | 0.720 | -0.41 | 0.59 |  |
| Rajasthan | 153.73 | 154.51 | 0.78 | 0.27 | 0.003 | 0.26 | 1.30 | 153.59 | 154.57 | 0.98 | 0.25 | 0.001 | 0.48 | 1.48 |  |
| Sikkim | 149.84 | 150.70 | 0.87 | 0.45 | 0.057 | -0.03 | 1.76 | 150.19 | 151.18 | 0.99 | 0.39 | 0.011 | 0.23 | 1.76 |  |
| Tamil Nadu | 151.68 | 153.24 | 1.56 | 0.30 | 0.001 | 0.97 | 2.14 | 151.38 | 152.28 | 0.90 | 0.25 | 0.001 | 0.42 | 1.39 |  |
| Tripura | 148.83 | 149.77 | 0.94 | 0.45 | 0.039 | 0.05 | 1.83 | 149.31 | 149.60 | 0.30 | 0.33 | 0.379 | -0.37 | 0.96 |  |
| Uttar Pradesh | 150.15 | 150.75 | 0.59 | 0.18 | 0.001 | 0.23 | 0.96 | 150.38 | 150.74 | 0.36 | 0.16 | 0.021 | 0.05 | 0.67 |  |
| Uttarakhand | NA | 152.85 | NA | NA | NA | NA | NA | NA | 152.69 | NA | NA | NA | NA | NA |  |
| West Bengal | 149.69 | 150.72 | 1.03 | 0.23 | 0.001 | 0.57 | 1.49 | 150.09 | 150.76 | 0.67 | 0.21 | 0.002 | 0.26 | 1.09 |  |
