# Supplementary material for: Trends of adult height in India from 1998 to 2015: Evidence from the National Family and Health Survey
Source: PLoS One. 2021 Sep 17;16(9):e0255676. doi: 10.1371/journal.pone.0255676 (PMC8448320; doi:10.1371/journal.pone.0255676)
Supplement: S10 Table — (DOCX) [file pone.0255676.s010.docx]

# Supportive information (S10 Table)

| **S10 Table State wise distribution of mean height of women according to age group, NFHS-4 and NFHS-3** | | | | | | | | | | | | | | | | |  |
| --- | --- | --- | --- | --- | --- | --- | --- | --- | --- | --- | --- | --- | --- | --- | --- | --- | --- |
|  | | 15 to 25 years | | | | | | | 26 to 50 Years | | | | | | | |  |
| **State** | **NFHS-4** | | **NFHS-3** | **Coef.** | **Robust Std. Err.** | **P-value** | **[95% Conf. Interval]** | | **NFHS-4** | **NFHS-3** | **Coef.** | **Robust Std. Err.** | **P- value** | **[95% Conf. Interval]** | |  |  |
| **India** | **151.83** | | **151.95** | **-0.12** | **0.06** | **0.051** | **-0.24** | **0.00** | **151.98** | **151.85** | **0.13** | **0.052** | **0.015** | **0.02** | **0.23** | | |
| Andaman and Nicobar islands | 152.31 | | NA | NA | NA | NA | NA | NA | 153.33 | NA | NA | NA | NA | NA | NA | | |
| Andhra Pradesh | 152.29 | | 152.01 | 0.28 | 0.20 | 0.157 | -0.11 | 0.68 | 151.84 | 151.54 | 0.31 | 0.158 | 0.053 | 0.00 | 0.62 | | |
| Arunachal Pradesh | 150.03 | | 150.41 | -0.38 | 0.27 | 0.162 | -0.91 | 0.15 | 151.17 | 151.00 | 0.16 | 0.259 | 0.526 | -0.34 | 0.67 | | |
| Assam | 150.58 | | 150.46 | 0.12 | 0.23 | 0.595 | -0.34 | 0.58 | 150.89 | 150.55 | 0.34 | 0.187 | 0.068 | -0.03 | 0.71 | | |
| Bihar | 149.89 | | 150.08 | -0.19 | 0.19 | 0.301 | -0.56 | 0.17 | 150.02 | 150.51 | -0.48 | 0.177 | 0.006 | -0.83 | -0.14 | | |
| Chandigarh | 153.93 | | NA | NA | NA | NA | NA | NA | 152.96 | NA | NA | NA | NA | NA | NA | | |
| Chhattisgarh | 151.28 | | 151.64 | -0.36 | 0.19 | 0.061 | -0.74 | 0.02 | 151.31 | 151.20 | 0.11 | 0.178 | 0.552 | -0.24 | 0.46 | | |
| Dadra and Nagar Haveli | 151.10 | | NA | NA | NA | NA | NA | NA | 151.75 | NA | NA | NA | NA | NA | NA | | |
| Daman and Diu | 153.15 | | NA | NA | NA | NA | NA | NA | 152.67 | NA | NA | NA | NA | NA | NA | | |
| Goa | 153.35 | | 152.87 | 0.48 | 0.46 | 0.289 | -0.41 | 1.38 | 152.36 | 152.43 | -0.07 | 0.347 | 0.841 | -0.75 | 0.61 | | |
| Gujarat | 152.95 | | 153.04 | -0.09 | 0.23 | 0.693 | -0.54 | 0.36 | 152.56 | 152.40 | 0.16 | 0.214 | 0.469 | -0.27 | 0.58 | | |
| Haryana | 153.97 | | 154.80 | -0.82 | 0.24 | 0.001 | -1.30 | -0.35 | 155.27 | 154.95 | 0.32 | 0.233 | 0.171 | -0.14 | 0.77 | | |
| Himachal Pradesh | 154.28 | | 153.88 | 0.40 | 0.26 | 0.126 | -0.11 | 0.92 | 153.89 | 153.60 | 0.29 | 0.203 | 0.157 | -0.11 | 0.69 | | |
| Jammu and Kashmir | 155.01 | | 154.96 | 0.04 | 0.29 | 0.880 | -0.53 | 0.62 | 155.37 | 154.26 | 1.11 | 0.227 | 0.001 | 0.66 | 1.55 | | |
| Jharkhand | 149.61 | | 149.68 | -0.07 | 0.26 | 0.800 | -0.57 | 0.44 | 149.87 | 150.03 | -0.16 | 0.194 | 0.406 | -0.54 | 0.22 | | |
| Karnataka | 152.47 | | 152.74 | -0.27 | 0.23 | 0.241 | -0.73 | 0.18 | 152.75 | 152.57 | 0.18 | 0.185 | 0.334 | -0.18 | 0.54 | | |
| Kerala | 154.82 | | 154.09 | 0.73 | 0.27 | 0.007 | 0.20 | 1.27 | 155.12 | 152.61 | 2.51 | 0.214 | 0.001 | 2.09 | 2.93 | | |
| Lakshadweep | 153.12 | | NA | NA | NA | NA | NA | NA | 152.70 | NA | NA | NA | NA | NA | NA | | |
| Madhya Pradesh | 152.33 | | 152.44 | -0.11 | 0.19 | 0.561 | -0.49 | 0.27 | 152.10 | 152.75 | -0.64 | 0.140 | 0.001 | -0.92 | -0.37 | | |
| Maharashtra | 152.14 | | 152.41 | -0.27 | 0.22 | 0.221 | -0.69 | 0.16 | 151.86 | 151.64 | 0.22 | 0.156 | 0.163 | -0.09 | 0.52 | | |
| Manipur | 152.24 | | 151.99 | 0.26 | 0.19 | 0.189 | -0.13 | 0.64 | 152.14 | 151.86 | 0.28 | 0.158 | 0.078 | -0.03 | 0.59 | | |
| Meghalaya | 148.36 | | 148.15 | 0.21 | 0.38 | 0.587 | -0.54 | 0.95 | 149.29 | 149.42 | -0.13 | 0.330 | 0.700 | -0.77 | 0.52 | | |
| Mizoram | 152.77 | | 151.62 | 1.15 | 0.33 | 0.001 | 0.51 | 1.80 | 152.34 | 151.77 | 0.57 | 0.267 | 0.034 | 0.04 | 1.09 | | |
| Nagaland | 152.63 | | 152.20 | 0.43 | 0.27 | 0.114 | -0.10 | 0.97 | 153.23 | 152.93 | 0.30 | 0.236 | 0.204 | -0.16 | 0.76 | | |
| Delhi | 152.50 | | 153.31 | -0.81 | 0.49 | 0.102 | -1.78 | 0.16 | 151.70 | 154.16 | -2.46 | 0.451 | 0.001 | -3.35 | -1.58 | | |
| Odisha | 150.56 | | 150.88 | -0.32 | 0.21 | 0.127 | -0.73 | 0.09 | 150.82 | 150.97 | -0.15 | 0.178 | 0.408 | -0.50 | 0.20 | | |
| Puducherry | 153.57 | | NA | NA | NA | NA | NA | NA | 153.17 | NA | NA | NA | NA | NA | NA | | |
| Punjab | 154.90 | | 154.76 | 0.14 | 0.25 | 0.573 | -0.35 | 0.63 | 155.82 | 154.68 | 1.14 | 0.199 | 0.001 | 0.74 | 1.53 | | |
| Rajasthan | 154.22 | | 154.51 | -0.29 | 0.22 | 0.199 | -0.73 | 0.15 | 154.52 | 154.57 | -0.05 | 0.225 | 0.835 | -0.49 | 0.39 | | |
| Sikkim | 151.96 | | 150.70 | 1.25 | 0.39 | 0.001 | 0.49 | 2.02 | 153.06 | 151.18 | 1.88 | 0.305 | 0.001 | 1.28 | 2.48 | | |
| Telangana | 152.50 | | NA | NA | NA | NA | NA | NA | 151.74 | NA | NA | NA | NA | NA | NA | | |
| Tamil Nadu | 152.59 | | 153.24 | -0.65 | 0.27 | 0.017 | -1.19 | -0.12 | 152.61 | 152.28 | 0.33 | 0.219 | 0.132 | -0.10 | 0.76 | | |
| Tripura | 149.46 | | 149.77 | -0.31 | 0.31 | 0.328 | -0.92 | 0.31 | 149.65 | 149.60 | 0.05 | 0.257 | 0.852 | -0.46 | 0.55 | | |
| Uttar Pradesh | 150.84 | | 150.75 | 0.09 | 0.12 | 0.462 | -0.15 | 0.33 | 150.70 | 150.74 | -0.05 | 0.112 | 0.685 | -0.27 | 0.17 | | |
| Uttarakhand | 152.47 | | 152.85 | -0.37 | 0.24 | 0.117 | -0.84 | 0.09 | 152.83 | 152.69 | 0.14 | 0.217 | 0.512 | -0.28 | 0.57 | | |
| West Bengal | 150.94 | | 150.72 | 0.22 | 0.17 | 0.199 | -0.12 | 0.56 | 150.93 | 150.76 | 0.16 | 0.169 | 0.340 | -0.17 | 0.49 | | |
