# Supplementary material for: Trends of adult height in India from 1998 to 2015: Evidence from the National Family and Health Survey
Source: PLoS One. 2021 Sep 17;16(9):e0255676. doi: 10.1371/journal.pone.0255676 (PMC8448320; doi:10.1371/journal.pone.0255676)
Supplement: S11 Table — (DOCX) [file pone.0255676.s011.docx]

# Supportive information (S11 Table)

| **S11 Table State wise distribution of mean height of men according to age group, NFHS-4 and NFHS-3** | | | | | | | | | | | | | | | |
| --- | --- | --- | --- | --- | --- | --- | --- | --- | --- | --- | --- | --- | --- | --- | --- |
|  | **15 to 25 years** | | | | | | | **26 to 50 Years** | | | | | | | |
| **State** | **NFHS-4** | **NFHS-3** | **Coef.** | **Robust Std. Err.** | **P-value** | **[95% Conf. Interval]** | | **NFHS-4** | **NFHS-3** | **Coef.** | **Robust Std. Err.** | **P-value** | **[95% Conf. Interval]** | |  |
| **India** | **163.38** | **164.48** | **-1.10** | **0.11** | **0.001** | **-1.31** | **-0.88** | 163.68 | **164.54** | **-0.86** | **0.09** | **0.001** | **-1.03** | **-0.69** |  |
| Andaman and Nicobar islands | 160.12 | NA | NA | NA | NA | NA | NA | 162.67 | NA | NA | NA | NA | NA | NA |  |
| Andhra Pradesh | 164.44 | 164.44 | 0.01 | 0.53 | 0.988 | -1.04 | 1.06 | 164.18 | 163.99 | 0.19 | 0.32 | 0.545 | -0.44 | 0.82 |  |
| Arunachal Pradesh | 159.04 | 160.64 | -1.60 | 0.65 | 0.015 | -2.88 | -0.32 | 160.27 | 161.84 | -1.57 | 0.47 | 0.001 | -2.50 | -0.64 |  |
| Assam | 161.36 | 162.65 | -1.29 | 0.56 | 0.022 | -2.40 | -0.19 | 161.88 | 163.03 | -1.15 | 0.36 | 0.001 | -1.85 | -0.45 |  |
| Bihar | 161.35 | 163.26 | -1.91 | 0.49 | 0.001 | -2.87 | -0.96 | 161.78 | 163.19 | -1.41 | 0.41 | 0.001 | -2.21 | -0.61 |  |
| Chandigarh | 168.27 | NA | NA | NA | NA | NA | NA | 165.78 | NA | NA | NA | NA | NA | NA |  |
| Chhattisgarh | 162.29 | 163.07 | -0.78 | 0.48 | 0.103 | -1.72 | 0.16 | 162.57 | 163.19 | -0.62 | 0.33 | 0.064 | -1.28 | 0.04 |  |
| Dadra and Nagar Haveli | 162.38 | NA | NA | NA | NA | NA | NA | 159.66 | NA | NA | NA | NA | NA | NA |  |
| Daman and Diu | 160.74 | NA | NA | NA | NA | NA | NA | 160.97 | NA | NA | NA | NA | NA | NA |  |
| Goa | 163.76 | 164.99 | -1.22 | 0.84 | 0.149 | -2.89 | 0.44 | 164.17 | 164.63 | -0.46 | 0.49 | 0.357 | -1.43 | 0.52 |  |
| Gujarat | 164.35 | 165.30 | -0.95 | 0.52 | 0.068 | -1.97 | 0.07 | 164.54 | 165.42 | -0.88 | 0.33 | 0.008 | -1.53 | -0.23 |  |
| Haryana | 164.90 | 167.65 | -2.76 | 0.48 | 0.001 | -3.71 | -1.81 | 166.42 | 168.07 | -1.65 | 0.40 | 0.000 | -2.43 | -0.87 |  |
| Himachal Pradesh | 166.97 | 165.78 | 1.19 | 0.64 | 0.066 | -0.08 | 2.45 | 166.36 | 165.30 | 1.06 | 0.43 | 0.015 | 0.21 | 1.91 |  |
| Jammu and Kashmir | 165.77 | 167.02 | -1.25 | 0.61 | 0.039 | -2.44 | -0.06 | 167.06 | 166.40 | 0.66 | 0.44 | 0.131 | -0.20 | 1.52 |  |
| Jharkhand | 160.38 | 162.12 | -1.74 | 0.56 | 0.002 | -2.83 | -0.64 | 160.49 | 162.48 | -2.00 | 0.47 | 0.001 | -2.93 | -1.07 |  |
| Karnataka | 162.56 | 164.68 | -2.11 | 0.49 | 0.001 | -3.08 | -1.15 | 163.09 | 165.13 | -2.04 | 0.36 | 0.001 | -2.76 | -1.33 |  |
| Kerala | 165.28 | 167.39 | -2.11 | 0.62 | 0.001 | -3.34 | -0.89 | 166.40 | 166.28 | 0.12 | 0.36 | 0.748 | -0.60 | 0.83 |  |
| Lakshadweep | 162.80 | NA | NA | NA | NA | NA | NA | 164.23 | NA | NA | NA | NA | NA | NA |  |
| Madhya Pradesh | 163.73 | 164.71 | -0.98 | 0.42 | 0.021 | -1.80 | -0.15 | 164.35 | 165.57 | -1.22 | 0.29 | 0.001 | -1.79 | -0.65 |  |
| Maharashtra | 163.33 | 164.79 | -1.46 | 0.47 | 0.002 | -2.38 | -0.53 | 163.24 | 164.77 | -1.53 | 0.33 | 0.001 | -2.19 | -0.88 |  |
| Manipur | 163.36 | 162.92 | 0.44 | 0.38 | 0.247 | -0.31 | 1.19 | 163.01 | 163.12 | -0.11 | 0.30 | 0.720 | -0.70 | 0.49 |  |
| Meghalaya | 157.37 | 155.81 | 1.57 | 0.89 | 0.079 | -0.18 | 3.32 | 158.91 | 158.39 | 0.51 | 0.65 | 0.432 | -0.78 | 1.80 |  |
| Mizoram | 163.28 | 162.33 | 0.95 | 0.72 | 0.184 | -0.46 | 2.36 | 162.25 | 161.95 | 0.30 | 0.46 | 0.513 | -0.61 | 1.21 |  |
| Nagaland | 162.82 | 161.55 | 1.27 | 0.54 | 0.020 | 0.20 | 2.34 | 162.82 | 162.98 | -0.16 | 0.43 | 0.709 | -1.01 | 0.69 |  |
| Delhi | 164.97 | 165.03 | -0.07 | 0.92 | 0.942 | -1.87 | 1.74 | 164.57 | 165.85 | -1.28 | 0.58 | 0.027 | -2.42 | -0.15 |  |
| Odisha | 161.70 | 162.74 | -1.05 | 0.45 | 0.019 | -1.92 | -0.17 | 162.16 | 162.91 | -0.75 | 0.36 | 0.036 | -1.45 | -0.05 |  |
| Puducherry | 163.06 | NA | NA | NA | NA | NA | NA | 162.27 | NA | NA | NA | NA | NA | NA |  |
| Punjab | 166.83 | 167.87 | -1.04 | 0.52 | 0.047 | -2.06 | -0.02 | 167.40 | 168.79 | -1.40 | 0.45 | 0.002 | -2.28 | -0.52 |  |
| Rajasthan | 165.20 | 166.08 | -0.88 | 0.44 | 0.047 | -1.74 | -0.01 | 165.92 | 167.13 | -1.21 | 0.35 | 0.001 | -1.90 | -0.52 |  |
| Sikkim | 158.87 | 159.77 | -0.91 | 1.07 | 0.398 | -3.03 | 1.21 | 159.98 | 159.43 | 0.56 | 0.67 | 0.410 | -0.78 | 1.89 |  |
| Telangana | 165.32 | NA | NA | NA | NA | NA | NA | 163.29 | NA | NA | NA | NA | NA | NA |  |
| Tamil Nadu | 163.04 | 164.94 | -1.90 | 0.43 | 0.001 | -2.75 | -1.05 | 164.85 | 164.32 | -1.03 | 0.30 | 0.001 | -1.63 | -0.43 |  |
| Tripura | 160.78 | 161.68 | -0.91 | 0.63 | 0.152 | -2.15 | 0.34 | 161.39 | 161.17 | 0.22 | 0.51 | 0.669 | -0.80 | 1.24 |  |
| Uttar Pradesh | 163.13 | 163.83 | -0.70 | 0.22 | 0.001 | -1.12 | -0.28 | 163.35 | 164.18 | -0.83 | 0.18 | 0.001 | -1.18 | -0.48 |  |
| Uttarakhand | 163.39 | 164.45 | -1.05 | 0.60 | 0.078 | -2.23 | 0.12 | 163.95 | 164.82 | -0.86 | 0.41 | 0.038 | -1.68 | -0.05 |  |
| West Bengal | 162.33 | 163.53 | -1.20 | 0.45 | 0.008 | -2.09 | -0.31 | 162.19 | 162.71 | -0.52 | 0.30 | 0.081 | -1.11 | 0.06 |  |
