# Supplementary material for: Trends of adult height in India from 1998 to 2015: Evidence from the National Family and Health Survey
Source: PLoS One. 2021 Sep 17;16(9):e0255676. doi: 10.1371/journal.pone.0255676 (PMC8448320; doi:10.1371/journal.pone.0255676)
Supplement: Attachment — Submitted filename: Comments and suggestions for authors_PLOSE ONE_D_21_05575.docx [file pone.0255676.s012.docx]

**Comments and suggestions for authors**

I would like to appreciate authors for this intensive research work and robust analysis of nationally representative data on the most discussed topic such as “Trends of adult height in India from 1998 to 2015: Evidence from the National Family and Health Survey.” The presentation of the data seems empirically strong and the paper is generally well written, with an appropriate focus. This study may be accepted for publication in the journal with reviewer following suggestions and comments for further improvements.

1. The authors can incorporate the Limitations of the study in the separate section of the manuscript.
