# Supplementary material for: Trends of adult height in India from 1998 to 2015: Evidence from the National Family and Health Survey
Source: PLoS One. 2021 Sep 17;16(9):e0255676. doi: 10.1371/journal.pone.0255676 (PMC8448320; doi:10.1371/journal.pone.0255676)
Supplement: Attachment — Submitted filename: Response to Reviewers.docx [file pone.0255676.s013.docx]

| Comments | Response |
| --- | --- |
| #Reviewer 1 |  |
| 1. The authors can incorporate the Limitations of the study in the separate section of the manuscript. | A separate section on ‘Limitations’ have been added after ‘Conclusion’ |
| #Reviewer 2 |  |
| 1. Introduction: Much of introduction focused on how genetic factors contribute to the height of population. However, other social-demographic and nutrition role in determining the height is not well explained. Further, authors must address to provide the research gap. | The role of socio-demographic factors and nutrition in determining height has been incorporated in the introduction section (3^rd^ and 4^th^ paragraph in the introduction section)  Research gap has also been addressed in the introduction section (in the penultimate paragraph of the introduction section) |
| 2. Methods: Authors must provide the sampling strategy employed by the NFHS. Also give more detailed information about how height of adult population has been measured. Further, provide the delayed clarification if any different methods used to measure the height in different rounds of NFHS survey. | The information sought has been incorporated within the two paragraphs of the Methodology section. |
| 3. Discussion: Discussion section is need to be strengthened with current literature on height of population with regards to various biological and social studies. Also, write a separate section on conclusion. | Two new paragraphs have been incorporated in the discussion sections to address the points as suggested. A separate section on Conclusion has been included. |
